# Estimating the Sodium Content: A Case Series of Benign and Malignant Renal Tumours Using ^23^Na‐MRI at 3 T

**DOI:** 10.1002/nbm.70338

**Published:** 2026-06-24

**Authors:** Ines Horvat‐Menih, Jonathan R. Birchall, Maria J. Zamora‐Morales, Alice Bebb, Joshua D. Kaggie, Frank Riemer, Andrew B. Gill, Andrew N. Priest, Marta Wylot, Iosif A. Mendichovszky, Anne Y. Warren, James Jones, James N. Armitage, Thomas J. Mitchell, Grant D. Stewart, Mary A. McLean, Ferdia A. Gallagher

**Affiliations:** ^1^ Department of Radiology University of Cambridge Cambridge UK; ^2^ Mohn Medical Imaging and Visualization Centre, Department of Radiology Haukeland University Hospital Helse Bergen Bergen Norway; ^3^ Department of Radiology Royal Papworth Hospital Cambridge UK; ^4^ Department of Radiology Addenbrooke's Hospital, Cambridge University Hospitals NHS Foundation Trust Cambridge UK; ^5^ Department of Pathology Addenbrooke's Hospital, Cambridge University Hospitals NHS Foundation Trust Cambridge; ^6^ Department of Oncology Addenbrooke's Hospital, Cambridge University Hospitals NHS Foundation Trust Cambridge UK; ^7^ Department of Urology Addenbrooke's Hospital, Cambridge University Hospitals NHS Foundation Trust Cambridge UK; ^8^ Department of Surgery University of Cambridge Cambridge UK

**Keywords:** magnetic resonance imaging, oncocytoma, renal cell carcinoma, sodium concentration, T2 relaxation

## Abstract

Accurate and non‐invasive subtyping of localised renal tumours is an important unmet clinical challenge in uro‐oncology and has significant implications for patient mortality and quality of life. Developing novel imaging methods to characterise and stratify indeterminate kidney tumours at an early stage has the potential to address this clinical challenge. Here we applied sodium MRI (^23^Na‐MRI) to estimate kidney tumour sodium content in a prospectively recruited case series of 10 patients (mean age ± SD 64 ± 8 years; 7:3 male:female ratio). The patients had localised renal tumours which included six renal oncocytomas (ROs), two chromophobe renal cell carcinomas (chRCCs), three clear cell RCCs (ccRCC) and one papillary RCC (pRCC). The patients underwent ^23^Na‐MRI at 3 T (3D sodium cones and double‐angle B_1_ mapping) and ^1^H‐MRI which included *R*_2_* mapping and intravoxel incoherent motion (IVIM) diffusion weighted imaging (DWI). The following imaging parameters were quantified within the renal tumours and in the normal‐appearing kidney parenchyma: apparent total sodium concentration (TSC); apparent ^23^Na and ^1^H relaxation rates (*R*_2_*); perfusion fraction (*f*_p_); and diffusion coefficient (*D*_t_). ^23^Na‐MRI findings were correlated with conventional ^1^H‐MRI measures of perfusion, hypoxia and cellularity. The mean apparent TSC in ccRCC and in pRCC were 135 ± 59 mM and 81 mM, respectively. The apparent TSC was significantly higher in ROs compared to chRCCs: 162 ± 58 mM vs. 71 ± 2 mM (*p* < 0.05). The apparent TSC inversely correlated with ^1^H‐*R*_2_* (Spearman r = −0.39, *p* < 0.05). In conclusion, this study showed the feasibility and potential of using ^23^Na‐MRI in renal tumours to probe sodium concentrations. These preliminary findings suggest a differential sodium content between benign ROs and malignant chRCCs. The inverse correlation between sodium concentration and ^1^H‐*R*_2_* as a surrogate of hypoxia may indicate a biophysical relationship between the two which requires further validation in larger patient cohorts.

## Graphical abstract

^23^Na‐MRI at 3 T was applied to estimate TSC in patients with kidney tumours of different pathologies, which are indistinguishable on conventional imaging methods. The apparent TSC was significantly higher in renal oncocytomas (benign) compared to chromophobe renal cell carcinomas (malignant): 162 ± 58 mM vs. 71 ± 2 mM (*p* < 0.05). These preliminary findings suggest a differential sodium content between benign and malignant kidney tumours and could guide development of imaging methods to address this clinical challenge.

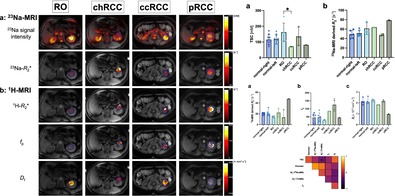

**Abbreviations**
CAIX: carbonic anhydrase 9
ccLS: clear cell likelihood score
ccRCC: clear cell renal cell carcinoma
chRCC: chromophobe renal cell carcinoma
ECOG: Eastern Cooperative Oncology Group
IBM‐Renal: Investigation of the differential biology of Benign and Malignant renal masses using advanced MRI techniques
IVIM DWI: intravoxel incoherent motion diffusion weighted imaging
NHE1: sodium hydrogen exchanger 1
pRCC: papillary renal cell carcinoma
RCC: renal cell carcinoma
RO: renal oncocytoma
TSC: total sodium concentration

## 1 Introduction

Renal tumours pose an increasing health economic burden due to the rising incidental detection of localised renal masses, which present a diagnostic dilemma as they often remain indeterminate on initial evaluation due to the low specificity of current clinical imaging methods [1]. This diagnostic uncertainty may result in unnecessary surgery, with the potential loss in renal function for those patients undergoing inappropriate nephrectomy. Conversely, a long‐term active surveillance strategy with imaging may be more appropriate than surgical resection for many patients, but this can affect patient mortality and morbidity if the detection of a malignant tumour is delayed and equally can result in psychological morbidity for patients with benign disease as they await a diagnosis [2]. Renal mass biopsy prior to the decision for surgery can aid in the identification of benign cases, but misdiagnosis may result due to the challenges of accurately sampling small lesions, particularly in the context of tumour heterogeneity [3]. Novel non‐invasive imaging techniques could aid the characterisation of renal masses on a whole‐tumour level.

Functional MRI methods enable tumour biology to be probed in addition to morphological evaluation and therefore could facilitate differentiation and stratification, as exemplified by the recently developed clear cell likelihood scoring system (ccLS) [4]. Although the ccLS requires qualitative interpretation by a radiologist, quantitative MRI biomarkers have the potential of providing objective, robust, quantitative comparisons between patients [5]. Despite the potential of these functional MRI metrics for clinical benefit, there have been few published studies in this field [6, 7], which have mostly reported surrogates for perfusion [8], cellularity [9] and hypoxia [10], and require further validation. For example, diffusion‐weighted imaging (DWI) in the kidney captures an interplay of both tubular and vascular components and therefore provides a non‐specific and complex biomarker of cellularity similar to that in other organs [11, 12].

Regulation of plasma and urinary sodium concentration is a primary renal function, but the role of sodium and its concentration gradient within kidney tumours is not well understood [13, 14]. Many cancers exhibit alterations in ion gradients to maintain an alkaline intracellular pH and an acidic extracellular pH, which promotes survival and tumour aggressiveness [15]. Furthermore, it has been suggested that activation of the sodium hydrogen exporter (NHE1) can be the initial step driving the development of other cancer hallmarks such as the Warburg effect, with a metabolic shift of cancer cells to preferentially using glycolytic pathways over oxidative ones and increased cell proliferation [16]. Therefore, detecting different tumour sodium phenotypes may provide insight into genetic drivers, as well as the hypoxic and pH characteristics of kidney tumours.

Sodium MRI (^23^Na‐MRI) enables the non‐invasive quantification of the spatial and temporal alterations in sodium concentrations [17, 18]. In oncological imaging, ^23^Na‐MRI has been shown to detect higher sodium concentrations in tumours compared to the normal‐adjacent prostate tissue [19] and in ovarian cancer the total sodium concentration has been shown to correlate negatively with cell density [20]. ^23^Na‐MRI can be used to detect the normal physiological corticomedullary sodium gradient, its response to diuretics [21], as well as changes in renal disease [22]. Here we set out to explore the role of ^23^Na‐MRI in renal mass characterisation and to assess sodium concentration in benign and malignant renal tumours. The underlying biological changes driving the sodium signal were explored by correlating the ^23^Na‐MRI signal with factors influencing sodium regulation in vivo, including deoxygenation, perfusion and diffusion, as probed with multiparametric MRI.

## 2 Materials and Methods

### 2.1 Ethics and Patient Recruitment

Consecutive patients with localised renal masses were prospectively recruited from a uro‐oncology clinic in a tertiary healthcare centre between January–April 2023 and signed the informed consent for the feasibility study approved by the institutional review board (**I**nvestigation of the differential biology of **B**enign and **M**alignant **Renal** masses using advanced MRI techniques, **IBM**‐**Renal**) [23]. The main inclusion criteria were: ≥18 years of age, clinical suspicion of a renal mass and an Eastern Cooperative Oncology Group (ECOG) performance status ≤ 1. Key exclusion criteria were lack of suitability for MRI, significant comorbidities and pregnancy. Histopathology of the renal mass was determined either on biopsy or on the surgical specimen, depending on the clinical management.

### 2.2 ^23^Na‐MRI Acquisition and Analysis

Recruited patients underwent ^23^Na‐MRI on a clinical 3 T scanner (Discovery MR750, GE Healthcare, Waukesha, Wisconsin), using a large field of view (FOV) ~ 48 cm ^23^Na‐tuned transmit/receive birdcage coil (Rapid Biomedical GmbH, Rimpar, Germany) [24]. High‐resolution sodium images were acquired with a 3D cones trajectory [25], using the following acquisition parameters, which were similar to other healthy volunteer studies using the same set‐up^26^: echo time (TE) 0.705 ms, repetition time (TR) 100 ms, flip angle 70°, nominal voxel size 4 × 4 × 8 mm, effective voxel size (defined as voxel size including the effects of point‐spread function (PSF), *T*_2_* blurring and processing) 8 × 8 × 16 mm, 1402 transients, 5 averages, receiver bandwidth 167 kHz and 11 min 41 s duration. A pair of 80 mM agar gel NaCl phantoms were placed within the imaging FOV and served as a calibration standard for signal intensity [26]. B_1_ maps were acquired in all patients, using the double angle method (DAM) as previously described [26, 27], using the following acquisition parameters: flip angles 40° and 80°, TR 150 ms, NEX 4, 197 transients and scan duration 2 min per flip. Low resolution 3D sodium cones images (TR 100 ms, flip angle 70°, nominal voxel size 9 × 9 × 9 mm, effective voxel size 16 × 16 × 16 mm, 197 transients, 4 averages, receiver bandwidth 125 kHz, 1 min 19 s duration per series) were acquired at six different echo times (TEs = 0.7, 1.5, 2, 4, 8, 16 ms) to quickly estimate the sodium *T*_2_* values using our established methodology [26] to explore whether there were interesting differences across kidney tumour subtypes. Anatomical fat/water T_1_w proton MRI was acquired with a 3D two‐point Dixon sequence using the MR system‐integrated body coil, within a single breath hold: TEs 1.1/2.2 ms, TR 3.7 ms, flip angle 15°, 1 average, matrix size 256 × 192, 0.7 phase FOV and 40 cm FOV.

Maps of the uncorrected total sodium concentration (TSC) were generated relative to an 80 mM agar gel ^23^Na‐phantom placed within the same FOV using MATLAB version R2023b (MathWorks, Natick MA). Maps of monoexponential estimates of the ^23^Na *T*_2_* relaxation time were generated from linear fitting of log‐transformed intensities of ^23^Na signal in the variable‐TE series. Regions of interest (ROIs) within tumours, normal‐appearing kidney parenchyma and liver were drawn on 2D axial slices in each parameter map by a resident in training with 4 years of experience in kidney segmentation, supervised by a consultant academic radiologist with more than 15 years of experience. Mean signal intensities within these ROIs were extracted. As an additional method for estimation of TSC, the ^23^Na signal from the liver was used as a reference by dividing the uncorrected TSC from each ROI with the TSC from the liver region within each patient, multiplying it by the mean liver TSC obtained from healthy volunteers (40 mM) [26]. Transmit and receive B_1_ uniformity correction was performed in each patient on a per‐region basis to obtain estimates of B_1_‐corrected TSC per region, as described by Birchall et al. [26]. The ^23^Na‐*R*_2_* relaxation rates, the reciprocal of *T*_2_* relaxation time, were calculated from the extracted apparent ^23^Na *T*_2_* values to compare these to the *R*_2_* maps from the ^1^H‐MRI.

To evaluate the effective resolution and PSF of the two cones trajectories employed in this work, a phantom experiment was conducted, as described in the Supporting Information and Figure S1.

### 2.3 ^1^H‐MRI Acquisition and Analysis

After the sodium acquisition with the birdcage transmit/receive coil, a change of coil (32‐channel cardiac array coil, GE Healthcare, Waukesha, WI) was necessary to perform proton *R*_2_* mapping and intravoxel incoherent motion (IVIM) DWI. *R*_2_* mapping was undertaken using the images acquired with the following parameters: FOV 40 cm, 12 echo times with 3.1 ms spacing, TR 110 ms, flip angle 30°, matrix size 256 × 224, slice thickness 4 mm, multiple breath holds, echo train length 12; and was processed as previously described [28]. IVIM DWI was acquired using a respiratory‐triggered dual spin‐echo echo‐planar imaging sequence and b‐values of 0, 10, 20, 30, 100, 300, 500, 700 and 900 s/mm^2^ with the following parameters: TE ~ 80 ms, TR 1 respiratory cycle, FOV 28.8 cm, slice thickness 4 mm, acquisition matrix 96 × 96, 2 averages for b‐values < 100 s mm^−2^ and 4 averages for higher b‐values, receiver bandwidth ± 111 kHz, parallel imaging (ASSET: Array coil Spatial Sensitivity Encoding) factor 2, averaged 3 directions, acquisition time 91 breaths (~10 min). MATLAB was used for processing of the IVIM DWI data to extract the perfusion fraction, *f*_p_ and the diffusion coefficient, *D*_t_, as previously described [28].

OsiriX MD v.14.0 (Pixmeo Sarl, Bernex, Switzerland) software was used to reorient and register the proton maps in the same space as the ^23^Na‐MRI acquisition and quantification of the mean values for individual parameters was performed using the same ROIs as annotated for ^23^Na‐MRI analysis, under the guidance of the same resident in‐training with 4 years of experience in kidney segmentation, supervised by a consultant radiologist as mentioned above.

### 2.4 Statistical Analysis

Statistical analysis was performed in GraphPad Prism v.10. Normality of the data was tested using the Shapiro–Wilk test. Normally distributed data and results are presented as mean (± standard deviation, S.D.) and non‐parametric data were presented as median (range). Welch's unpaired *t*‐test and Welch's ANOVA test were used for comparison of normally distributed imaging parameters across the kidney tumour subtypes and for nonparametric parameters, the Kruskal–Wallis with Dunn's multiple comparisons test was used. To probe for correlations between imaging parameters, the Spearman correlation coefficients were calculated from all extracted ROIs. *p* < 0.05 was used as the cut‐off for significance. Sample size was determined based on the power calculation in the ethically approved protocol.

## 3 Results

### 3.1 Patient Characteristics

A summary of the patient characteristics is shown in Table 1, with extended patient characteristics including data for each individual patient, as well as comorbidities, presented in Table S1. Ten patients with mean age ± SD = 64 ± 8 years were recruited, of which seven were men. As two patients had bilateral renal masses on MRI, the following subtypes were identified: six renal oncocytomas (RO), two chromophobe RCCs (chRCC), three clear cell RCCs (ccRCC) and one papillary RCC (pRCC). One patient had bilateral ROs and another presented with a right‐sided ccRCC and left‐sided chRCC. Initial detection of the renal mass was incidental in all participants. The mean tumour size at detection was 4.4 ± 1.6 cm. Four patients were on active surveillance, while six patients underwent nephrectomy shortly after their research imaging, including a patient with bilateral tumours where the right‐sided ccRCC was removed leaving the left‐sided chRCC to be followed up with active surveillance.

**TABLE 1:** Patient characteristics.

Age at detection (mean ± SD) [years]	64 ± 8
Sex (Female: male)	3:7
Presentation at detection (Incidental/symptom‐triggered investigation)	10/0
Tumour size at detection (mean ± SD) [cm]	4.4 ± 1.6
Histology	
RO	5 (+ 1 bilateral)
chRCC	2
ccRCC	3
pRCC	1
Clinical management	
Active surveillance	4
Nephrectomy	6

Abbreviations: ccRCC = clear cell RCC, chRCC = chromophobe RCC, pRCC = papillary RCC, RCC = renal cell carcinoma, RO = renal oncocytoma, SD = standard deviation.

### 3.2 ^23^Na‐MRI: Apparent TSC and *R*_2_* Quantification Across Kidney Tumour Subtypes

**FIGURE 1:**
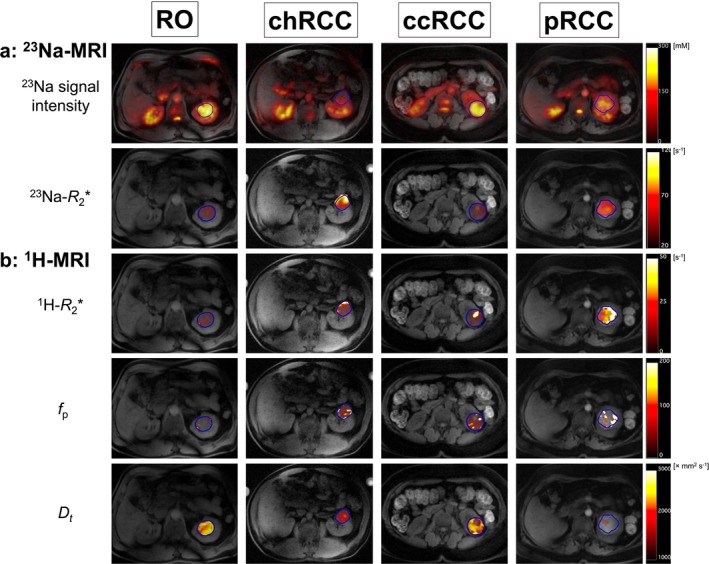
Representative parameter maps derived from: (a) ^23^Na‐MRI, including ^23^Na signal intensity and ^23^Na‐*R*_2_*; and (b) ^1^H‐MRI, including ^1^H‐*R*_2_*, *f*_p_ and *D*_t_; overlaid on T1w images for the evaluated kidney tumour subtypes as annotated. Abbreviations: ccRCC = clear cell RCC, chRCC = chromophobe RCC, pRCC = papillary RCC, RCC = renal cell carcinoma, RO = renal oncocytoma.

Representative maps of all extracted imaging parameters are shown in Figure 1. Sodium signal intensity maps overlaid on T_1_w anatomical images in examples of the evaluated kidney tumour subtypes are shown in Figure 1 (top row).

**FIGURE 2:**
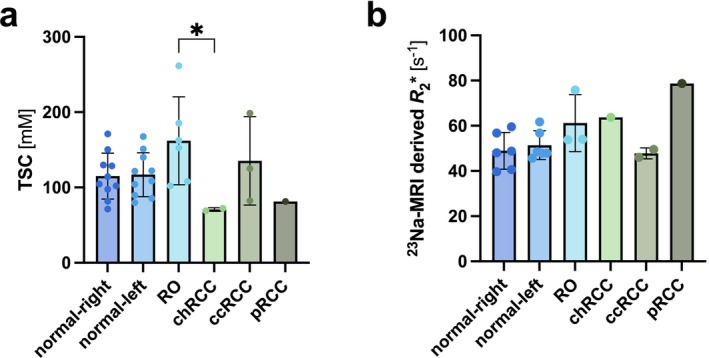
Barplots representing the B_1_‐corrected mean: (a) TSC; and (b) *R*_2_* in normal kidney parenchyma and across the kidney tumour subtypes. Welch's ANOVA test presented as *p < 0.05.

B_1_‐corrected, phantom‐referenced apparent TSC results across kidney tumour subtypes are depicted in Figure 2a, with TSC quantifications summarised in Table S2. The mean apparent TSC of the normal kidney parenchyma was similar bilaterally (right: 115 ± 31 mM; left: 117 ± 29 mM) with the highest apparent TSC observed in the benign ROs (162 ± 58 mM), followed by the ccRCCs (135 ± 59 mM), with the lowest apparent TSC measured in the chRCCs (71 ± 2 mM), followed by the pRCC (81 mM). The comparison between RO and chRCC was found to be statistically significant (*p* < 0.05). As depicted in the Figure S2, the apparent TSC quantifications referenced to liver showed similar trends to those observed using the phantom calibration only; however, the difference between RO and chRCC did not reach statistical significance by a small margin (Kruskal–Wallis with Dunn's multiple comparisons correction, *p* = 0.08).

*R*_2_* mapping of ^23^Na was performed in 6 patients; Figure 2b shows the comparison of the apparent *R*_2_* across kidney tumour subtypes, with the full results in Table S3. Interestingly, the trend for ^23^Na‐MRI derived apparent *R*_2_* was different compared to the apparent TSC results, with the highest values detected in the pRCC (79 s^−1^), followed by the chRCCs (64 s^−1^), the ROs (61 ± 13 s^−1^) and the ccRCCs (48 ± 2 s^−1^). Apparent *R*_2_* values obtained from normal kidney parenchyma were similar bilaterally (right: 49 ± 8 s^−1^, left: 51 ± 6 s^−1^). ANOVA analysis was not applied due to single data points for chRCC and pRCC, while no statistical significance was observed in Welch's *t*‐test comparing RO and ccRCC (*p* = 0.20).

### 3.3 Comparison of *R*_2_* Values Derived From ^23^Na‐MRI and ^1^H‐MRI

**FIGURE 3:**
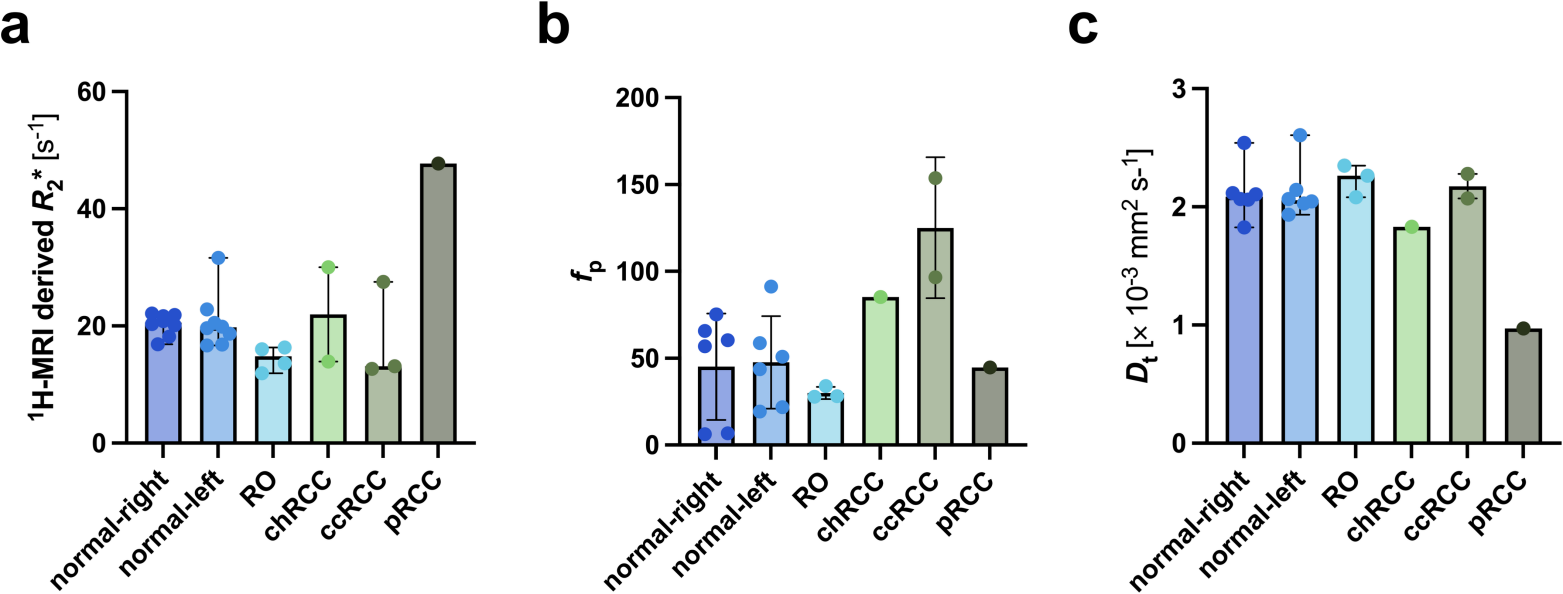
Barplots depicting ^1^H‐MRI results including: (a) *R*_2_* [median (range)]; (b) *f*_p_ [mean ± SD]; and (c) *D*_t_ [median (range)] across kidney tumour subtypes and normal parenchyma.

^23^Na‐MRI derived apparent *R*_2_* values were compared to those derived from ^1^H‐MRI, with representative images from different kidney tumour subtypes shown in Figure 1 (second and third rows). Table S3 summarises the quantitative results from the patients who underwent *R*_2_* mapping. The absolute values were of different magnitudes ranging between 40–79 s^−1^ for ^23^Na‐MRI derived *R*_2_* (Figure 2b) and between 12–48 s^−1^ for ^1^H‐MRI derived *R*_2_* (Figure 3a).

The highest observed apparent *R*_2_* values were observed in the single pRCC tumour on both ^1^H‐ and ^23^Na‐MRI. Similarly, the ccRCC tumour subtype exhibited the lowest *R*_2_* values using both techniques.

The largest difference between the apparent *R*_2_* values derived from the two nuclei was identified in the RO group: ^23^Na‐MRI derived *R*_2_* in this tumour type was among the highest (61 ± 13 s^−1^), while *R*_2_* derived from ^1^H‐MRI was among the lowest (15, range 12–16 s^−1^). Kruskal–Wallis with Dunn's multiple comparisons test applied to the ^1^H‐MRI derived *R*_2_* data across tumour subtypes was not statistically significant (*p* = 0.34).

### 3.4 IVIM DWI Results

IVIM DWI was acquired in the same 6 patients as the *R*_2_* mapping and representative maps are shown in Figure 1 (two bottom rows). Figure 3b,c represents the quantitative results for *f*_p_ and *D*_t_ respectively, comparing different kidney tumour subtypes, with detailed results found in Table S4.

The lowest *f*_p_ and the highest *D*_t_ were recorded in the RO group: 0.031 (± 0.004) and 2.264 (2.083–2.348) × 10^−3^ mm^2^ s^−1^, respectively. The ccRCC showed the highest *f*_p_ (0.125 ± 0.041), whereas pRCC exhibited the lowest *D*_t_ (0.970 × 10^−3^ mm^2^ s^−1^). However, none of the comparisons were statistically significant (*f*_p_: Welch's *t*‐test, *p* = 0.18; *D*_t_: Kruskal–Wallis with Dunn's multiple comparisons test, *p* = 0.30).

### 3.5 Correlations Between Imaging Parameters

**FIGURE 4:**
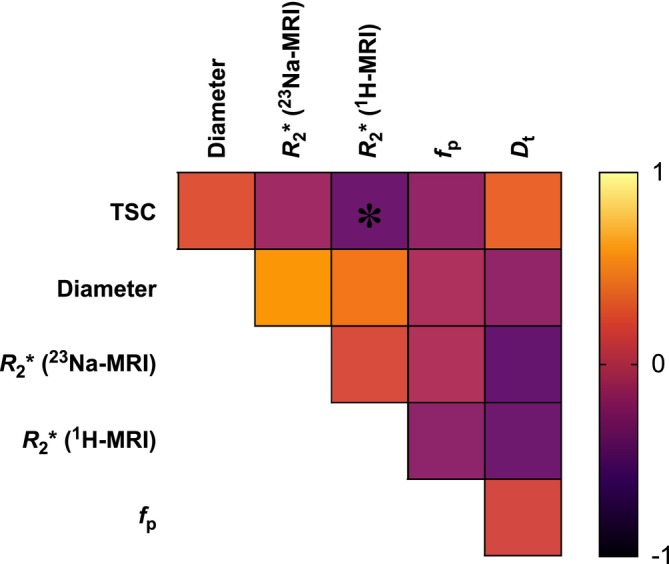
Correlation matrix of the imaging parameters, extracted from all analysed ROIs. Spearman correlation significance presented as *p < 0.05.

To understand the relationship between the tumour microenvironment on the TSC, a correlative analysis was performed with the correlation matrix shown in Figure 4.

TSC showed a significant negative correlation with the ^1^H‐derived *R*_2_* (Spearman r = −0.39, *p* < 0.05), but not with the ^23^Na‐derived apparent *R*_2_*. The correlation between both *R*_2_* parameters was not found to be statistically significant (Spearman r = 0.17, *p* = 0.50) and no other correlations were significant.

## 4 Discussion

This is the first report to measure the sodium concentration in renal tumours using ^23^Na‐MRI, with previous studies focusing on the role of the technique in diffuse kidney disease [29, 30]. The quantified apparent TSC values within the normal kidney parenchyma in this study (115 ± 31 mM and 117 ± 29 mM in the left and right kidneys, respectively) were similar to those reported previously: Grist et al. (2020) [21] reported the mean TSC in the normal kidney parenchyma to be 91–94 mM in 12 healthy volunteers (mean age: 30 ± 2 years) across two sites, with lower values in the cortex (70–72 mM) and higher values in the medulla (133–137 mM). Haneder et al. (2013) [31] similarly reported a mean sodium concentration of 59 mM in the cortex and 99 mM in the medulla, studying 50 healthy volunteers with a mean age of 29.2 years. On the other hand, Birchall et al. (2024) [26] performed a study on 19 healthy volunteers using the same protocol and set‐up as in the present patient cohort and found normal kidney TSC values to be 71 ± 9 mM and 79 ± 15 mM TSC in the right and left kidneys, respectively. The differences between our patient cohort and previous studies on healthy volunteers could arise due to inherently different characteristics of the studied cohorts, including an older age group and accompanying comorbidities in our patient cohort, as well as technical differences.

Even though the sample size was small, the results indicate that ^23^Na‐MRI may be useful for measuring differential sodium content between benign ROs and their malignant counterpart chRCCs: differentiating these two tumour types is a major clinical challenge due to their similarity on conventional imaging as well as on biopsy and emerging research imaging methods are unable to distinguish between the two [32]. The higher apparent TSC in RO compared to chRCC may be partly explained by lower cellularity and an increased extracellular volume fraction, measured as a higher *D*_t_ in the former, although this was not statistically significant. We have previously observed in ovarian cancer a similar inverse correlation between TSC and cellularity measured on histology [20].

We detected an inverse relationship between the apparent TSC and *R*_2_* measured with ^1^H‐MRI, but not with the ^23^Na‐MRI derived apparent *R*_2_*, suggesting that the two multinuclear *R*_2_* measurements are probing different aspects of biology. We hypothesise that this could be due to differences in the compartmentalisation of sodium within the tissue compared to free water, as well as the quadrupolar relaxation properties of sodium compared to protons [33]. Due to the sample size, we were not able to assess the correlation between apparent TSC with *D*_t_ in each of the tumour subtypes individually, although a previous study in the healthy human kidney found that the TSC correlated with neither apparent diffusion coefficient (ADC) nor with *R*_2_* [31]. Although *D*_t_ has been reported to have the highest diagnostic accuracy for differentiating benign and malignant renal tumours, in a recent meta‐analysis, the same report suggests that IVIM studies are currently limited and future studies are required to confirm this finding, as well as to investigate the relationship between the TSC and diffusion parameters [34].

The ^23^Na‐*R*_2_* and ^1^H‐*R*_2_* values that we reported within the normal kidneys agreed with existing literature. As reported by Birchall et al. (2024), the ^23^Na‐*T*_2_* values in kidneys were on average 23 ± 3 ms, which corresponds to a similar apparent ^23^Na‐*R*_2_* value obtained in the present study (*T*_2_* values here are ~20 ms for both right (1/49 s^−1^) and left (1/51 s^−1^) kidneys). A systematic review of renal blood oxygenation level dependent (BOLD) MRI has reported values of 30 s^−1^ in the medulla and 20 s^−1^ in the cortex of healthy kidneys, or 19–20 s^−1^ across the entire renal parenchyma, which are also similar to the values obtained in the present study [35]. However, this is the first report of ^23^Na‐*T*_2_* measurements in kidney tumours. In contrast, ^1^H‐*R*_2_* measurements have been reported in renal tumours [10]: Wu et al. (2015) [10] observed a higher ^1^H‐*R*_2_* in benign lesions compared to RCC, but did not report results for individual kidney tumour subtypes or for benign lesions. Choi et al. (2014) [36] observed the highest ^1^H‐*R*_2_* in chRCCs across the three RCC subtypes, which is in agreement with our ^1^H‐*R*_2_* measurements, but they did not compare this to benign lesions.

*R*_2_* as measured on ^1^H‐MRI is often used as a surrogate of hypoxia [37], but is also affected by other changes in tissue such as iron overload. Changes in sodium concentration may also be indirectly linked to hypoxia and oxidative phosphorylation as a result of both the relationship between sodium‐hydrogen exchange and pH and the energetic requirements for the transmembrane sodium‐potassium pump [15, 16]. The important role of pH in renal tumours is exemplified by the extracellular expression of carbonic anhydrase IX (CAIX) as a pH regulator [38], which has been demonstrated in vivo using PET as well as with immunohistochemistry in ccRCC [39]. Metabolic rewiring of renal cancer can be indirectly imaged using ^99^Tc‐sestamibi with single‐photon emission computed tomography (SPECT) which detects high mitochondrial density [40]: this is a characteristic of oncocytic neoplasms where defective mitochondria accumulate due to an impaired respiratory complex I, resulting in hypoxia [41]. However, these techniques involve ionising radiation and cannot differentiate between RO and chRCC with high certainty [42]. Therefore, an MRI‐based approach to discriminate these two offers a potential tool to address this clinical unmet need.

## 5 Limitations

This was a proof‐of‐concept feasibility study and therefore the small patient cohort is a limitation. The low spatial resolution prevented differentiation of the renal medulla and cortex as previously observed and therefore changes in the cortico‐medullary gradient could not be assessed [21, 22]. It would also be interesting to estimate differences in sodium concentrations across distinct intratumoral regions and correlate these to histological measures of aggressiveness, but this was limited due to the acquired spatial resolution [43]. Further biological insights could have been gained by estimating intracellular sodium concentrations [44], however, this requires long acquisition times, which is a challenge in a clinical setting. We have also not corrected for cystic or necrotic regions within the tumours which may have affected the TSC quantification, but have used overall ^1^H‐MRI derived biomarkers to corroborate the ^23^Na‐MRI results. Finally, to more efficiently capture the fast‐decaying ^23^Na signal, multi‐echo 3D radial trajectory with short readout duration (2 ms, compared to 33 ms employed here) may be worth pursuing, as demonstrated by Blunck et al. (2018) at 7 T in the brain [45]. However, application in the abdomen at routine clinical field strengths has not been demonstrated yet. Multiple quantum filtering (MQF) approaches have also seen application in the brain at 4 and 7 T [46, 47], but these techniques involve discarding a significant proportion of the total ^23^Na signal and signal to noise ratios are unfortunately more limited in the abdomen due to the larger field‐of‐view of RF coils employed.

## 6 Conclusion

In conclusion, by detecting sodium concentrations within renal masses and by correlating the TSC with ^1^H‐*R*_2_*, we have shown the feasibility of using ^23^Na‐MRI to aid the characterisation of renal tumours and to differentiate benign from malignant disease. Future larger patient cohorts could evaluate whether the technique could be implemented as a routine imaging tool to non‐invasively characterise the entire tumour and whether this could better predict aggressiveness compared to a single biopsy alone.

## Author Contributions

Conceptualization: **Ines Horvat‐Menih**, **Jonathan R. Birchall**, **Iosif A. Mendichovszky**, **Anne Y. Warren**, **James Jones**, **James N. Armitage**, **Thomas J. Mitchell**, **Grant D. Stewart**, **Mary A. McLean**, **Ferdia A. Gallagher**. methodology: **Ines Horvat‐Menih**, **Jonathan R. Birchall**, **Joshua D. Kaggie**, **Frank Riemer**, **Mary A. McLean Andrew N. Priest**, **Andrew B. Gill**. software: **Jonathan R. Birchall**, **Mary A. McLean**. validation: **Ines Horvat‐Menih**, **Jonathan R. Birchall**, **Mary A. McLean**. formal analysis: **Ines Horvat‐Menih**, **Jonathan R. Birchall**, **Mary A. McLean**. investigation: **Ines Horvat‐Menih**, **Alice Bebb**. resources: **Ferdia A. Gallagher**, **Grant D. Stewart**. data curation: **Ines Horvat‐Menih**, **Alice Bebb**. writing – original draft preparation: **Ines Horvat‐Menih**. writing – review and editing: **Ines Horvat‐Menih**, **Jonathan R. Birchall**, **Maria J. Zamora‐Morales**, **Alice Bebb**, **Joshua D. Kaggie**, **Frank Riemer**, **Andrew B. Gill**, **Andrew N. Priest**, **M.W.,** **Iosif A. Mendichovszky**, **Anne Y. Warren**, **James Jones**, **James N. Armitage**, **Thomas J. Mitchell**, **Grant D. Stewart**, **Mary A. McLean Ferdia A. Gallagher**. visualization: **Ines Horvat‐Menih**, **Jonathan R. Birchall**. supervision: **James N. Armitage**, **Grant D. Stewart**, **Mary A. McLean Ferdia A. Gallagher**. project administration: **Ines Horvat‐Menih**, **Maria J. Zamora‐Morales**, **M.W.** funding acquisition: **Ferdia A. Gallagher**, **Grant D. Stewart**. All authors have read and agreed to the published version of the manuscript.

## Funding

This study was funded by Cancer Research UK, 10.13039/501100000289 (C19212/A27150, C19212/A29082, C19212/A16628, EDDPMA‐May22\100068), by the Mark Foundation for Cancer Research, 10.13039/100014599 (RG95043), Cancer Research UK Cambridge Institute, University of Cambridge, 10.13039/501100022011 (C9685/A25177 and CTRQQR‐2021\100012), the NIHR Cambridge Biomedical Research Centre, 10.13039/501100018956 (BRC‐1215‐20014 and NIHR203312). The authors have additional funding from the National Cancer Imaging Translational Accelerator (NCITA; C42780/A27066), the Cambridge Experimental Cancer Medicine Centre, the Mark Foundation Institute for Integrated Cancer Medicine (MFICM), and the Canadian Institute For Advanced Research (CIFAR).

## Disclosure

Horvat‐Menih I, Birchall JR, Zamora‐Morales MJ, et al. (2024). Quantitative sodium‐MRI detects differential sodium content in benign vs. malignant oncocytic renal tumours. Preprint at https://doi.org/10.1101/2024.06.19.24309026.

## Conflicts of Interest

G.D.S. has received educational grants from Pfizer, AstraZeneca and Intuitive Surgical; consultancy fees from Pfizer, MSD, EUSA Pharma and CMR Surgical; travel expenses from MSD and Pfizer; speaker fees from Pfizer; clinical lead (urology) National Kidney Cancer Audit and Topic Advisor for the NICE kidney cancer guideline. S.J.W. is a founder and director of Pinto Medical Consultancy. F.A.G. has research grants from GlaxoSmithKline and AstraZeneca, research support from GE Healthcare, and has consulted for AstraZeneca on behalf of the University of Cambridge.

## Supporting information


**Figure S1:** Line plots of ^23^Na signal intensity across a 5 mm NMR tube containing 0.9% NaCl in the L/R direction (a–b), A/P direction (c–d) and S/I direction (e–f). Axial images (g–h) acquired using both the nominal low‐resolution cones trajectory for DAM B_1_ mapping (left side) and nominal high‐resolution cones trajectory for TSC estimation (right side) are included for reference. Estimated FWHM values of the PSF are included in displays (a–f) for comparison.
**Table S1:** Extended patient characteristics. BMI = body mass index, ccRCC = clear cell renal cell carcinoma, chRCC = chromophobe renal cell carcinoma, F = female, M = male, pRCC = papillary renal cell carcinoma, RO = renal oncocytoma.
**Table S2:** TSC quantification results of kidney tumours and bilateral normal kidney parenchyma. RCC = renal cell carcinoma, TSC = total sodium concentration. Units of TSC are in mM.
**Figure S2:** Plot representing the B1‐corrected mean TSC from normal kidney parenchyma and across the kidney tumour subtypes, referenced to liver TSC signal.
**Table S3:** Quantification of R2* derived from 23Na‐MRI and 1H‐MRI in tumours and normal kidney parenchyma. Units: s−1.
**Table S4:** Quantification of IVIM DWI derived parameters fp (in arbitrary units) and Dt (in × 10−3 mm2 s−1) in tumours and normal kidney parenchyma.

## Data Availability

The data that support the findings of this study are available from the corresponding author upon reasonable request.
