# Supplementary material for: Estimating the Sodium Content: A Case Series of Benign and Malignant Renal Tumours Using 23Na‐MRI at 3 T
Source: NMR Biomed. 2026 Jun 24;39(8):e70338. doi: 10.1002/nbm.70338 (PMC13291926; doi:10.1002/nbm.70338)
Supplement: Supplementary file 1 — Figure S1: Line plots of 23Na signal intensity across a 5 mm NMR tube containing 0.9% NaCl in the L/R direction (a–b), A/P direction (c–d) and S/I direction (e–f). Axial images (g–h) acquired using both the nominal low‐resolution cones trajectory for DAM B1 mapping (left side) and nominal high‐resolution cones trajectory for TSC estimation (right side) are included for reference. Estimated FWHM values of the PSF are included in displays (a–f) for comparison. Table S1: Extended patient characteristics. BMI = body mass index, ccRCC = clear cell renal cell carcinoma, chRCC = chromophobe renal cell carcinoma, F = female, M = male, pRCC = papillary renal cell carcinoma, RO = renal oncocytoma. Table S2: TSC quantification results of kidney tumours and bilateral normal kidney parenchyma. RCC = renal cell carcinoma, TSC = total sodium concentration. Units of TSC are in mM. Figure S2: Plot representing the B1‐corrected mean TSC from normal kidney parenchyma and across the kidney tumour subtypes, referenced to liver TSC signal. Table S3: Quantification of R2* derived from 23Na‐MRI and 1H‐MRI in tumours and normal kidney parenchyma. Units: s−1. Table S4: Quantification of IVIM DWI derived parameters fp (in arbitrary units) and Dt (in × 10−3 mm2 s−1) in tumours and normal kidney parenchyma. [file NBM-39-e70338-s001.docx]

Estimating the Sodium Content: a Case Series of Benign and Malignant Renal Tumours using ^23^Na-MRI at 3 T

**Supplementary Methods**

To evaluate the effective resolution and point-spread function (PSF) of the two cones trajectories employed in this work, a phantom experiment was conducted using a sodium-tuned transmit/receive head coil (Rapid Biomedical GmbH, Rimpar, Germany) over the same 48 cm FOV and 120 x 120 x 120 reconstructed matrix size as employed in the main manuscript. A 5 mm diameter NMR tube containing 0.9% NaCl was positioned parallel to the bore of the magnet within the RF coil, chosen to approximate a point-source (in-plane nominal resolutions of 4 and 9 mm for the nominal high- and nominal low-resolution ^23^Na trajectories, respectively).

Image volumes were acquired with both trajectories using the parameters listed in the main manuscript. Line plots of the ^23^Na signal intensity through the centre of the phantom were created in both the L/R and A/P directions. The phantom was then rotated with the long axis left-right and a second pair of images were acquired to determine the ^23^Na signal intensity through the centre of the phantom in the S/I direction. Simulated images were also produced by creating a matrix of ones with dimensions matching that of the real raw data and performing reconstruction with the same parameters^1^. No temporal or spatial apodization were applied during reconstruction. A density compensation function was applied during the re-gridding of k-space from the cones trajectory to Cartesian space^2^. These real and simulated ^23^Na intensity plots are shown below in Supplementary Figure 1 a-f. Images through a central axial slice of the phantom acquired using the two trajectories are included in displays g-h for reference.


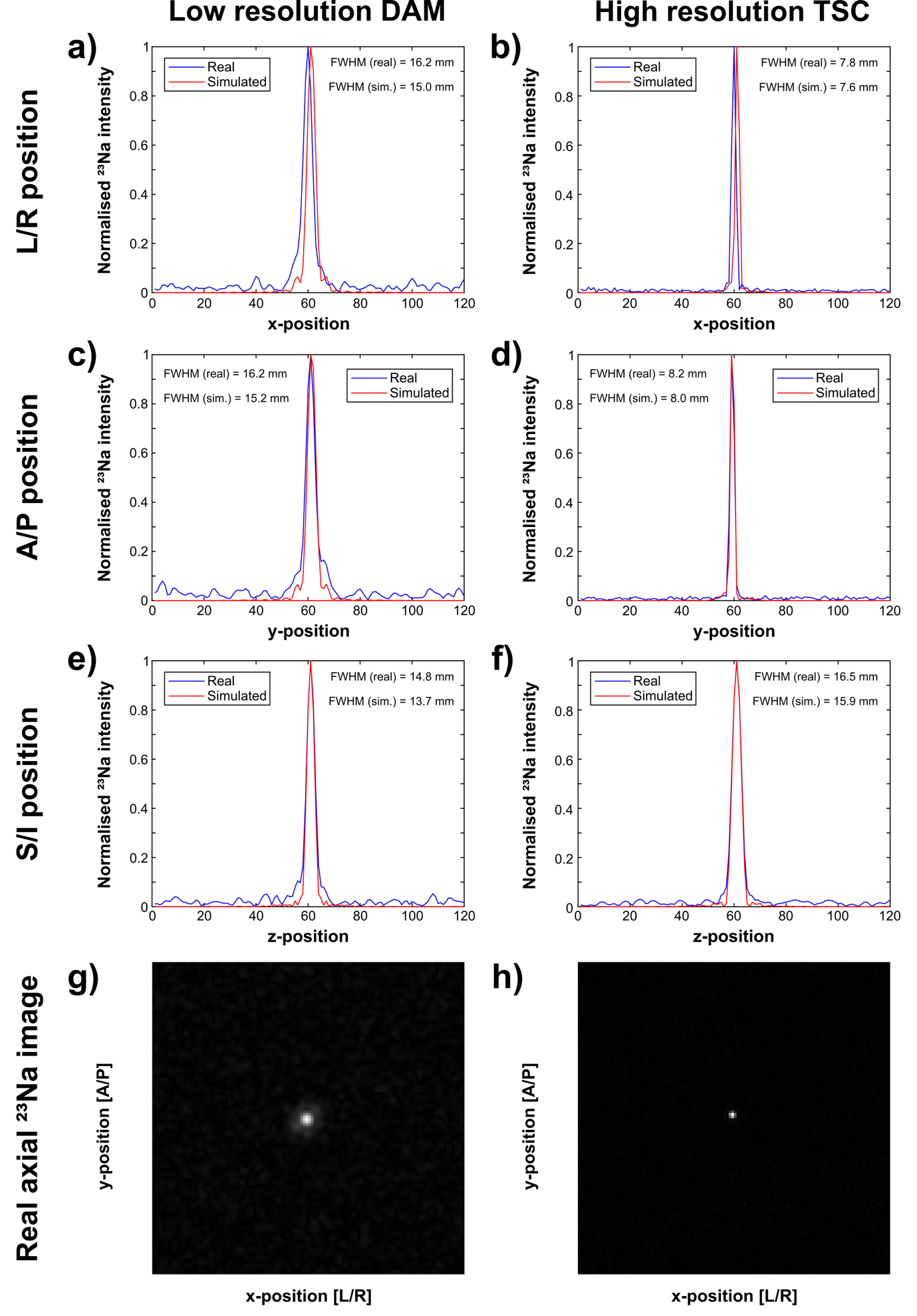


Supplementary Figure 1: Line plots of ^23^Na signal intensity across a 5 mm NMR tube containing 0.9% NaCl in the L/R direction (a-b), A/P direction (c-d) and S/I direction (e-f). Axial images (g-h) acquired using both the nominal low-resolution cones trajectory for DAM B_1_ mapping (left side) and nominal high-resolution cones trajectory for TSC estimation (right side) are included for reference. Estimated FWHM values of the PSF are included in displays (a-f) for comparison.

When considering real data, the FWHM of the ^23^Na signal intensity line plots was calculated to be ~16 mm for the nominal low-resolution cones trajectory (displays a,c,e) and ~8 mm in-plane for the nominal high-resolution cones trajectory (displays b,d). The corresponding FWHM of the ^23^Na signal intensity line plot through the phantom in the S/I direction (display f) was roughly double that of the other two directions (~ 16.5 mm) due to the two-fold lower nominal resolution of the high-resolution cones trajectory in this dimension (4 x 4 x 8 mm). These values are in good agreement with those we obtained via simulations (~8% larger for low-resolution cones and ~3% larger for high-resolution cones).

The observation of effective resolution roughly double that of the nominal resolution provides information on the effects of signal blurring due to the point-spread function of the trajectories and is consistent with a previous study observing voxel sizes 69 to 86% higher than nominal in high-resolution sodium imaging at 7 T^3^. It is possible that trajectories with shorter readout durations (e.g. 3D radial, FLORET) may confer advantages in this regard due to reduced *T*_2_* relaxation effects^4^.

1. Riemer, F. *et al.* Sodium (23Na) ultra-short echo time imaging in the human brain using a 3D-Cones trajectory. *Magn Reson Mater Phy* **27**, 35–46 (2014).

2. Zwart, N. R., Johnson, K. O. & Pipe, J. G. Efficient sample density estimation by combining gridding and an optimized kernel. *Magnetic Resonance in Med* **67**, 701–710 (2012).

3. Qian, Y., Zhao, T., Zheng, H., Weimer, J. & Boada, F. E. High‐resolution sodium imaging of human brain at 7 T. *Magnetic Resonance in Med* **68**, 227–233 (2012).

4. Polak, P., Schulte, R. F. & Noseworthy, M. D. An approach to evaluation of the point‐spread function for^23^ Na magnetic resonance imaging. *NMR in Biomedicine* **35**, e4627 (2022).

**Supplementary Data**

| Patient ID | Age  [years] | Sex | Presentation | Tumour diameter [cm] | Tumour side | Histology | Clinical management | Comorbidities | BMI  [kg/m^2^] |
| --- | --- | --- | --- | --- | --- | --- | --- | --- | --- |
| Patient 1 | 73 | M | Incidental | 3.0 | Left | RO | Active surveillance | History of colon cancer, duodenopancreatic neuroendocrine tumour, polymyalgia rheumatica, type 2 diabetes mellitus, ischaemic heart disease, hypertension, osteopenia, gastroesophageal reflux disease | 24.8 |
| Patient 2 | 73 | M | Incidental | 2.5 | Right | RO | Active surveillance | Hypertension | 24.7 |
| Patient 3 | 64 | M | Incidental | 7.1 | Right | RO | Active surveillance | Lewy body dementia, hypertension, | 21.8 |
| Patient 5  (tumour right) | 64 | M | Incidental | 5.0 | Bilateral | ccRCC | Nephrectomy | Hypercholesterolaemia, hypertension | 25.0 |
| Patient 5  (tumour left) | - | - | Incidental | 3.2 | - | chRCC | Active surveillance | - | - |
| Patient 6 | 65 | M | Incidental | 5.2 | Left | RO | Nephrectomy | Hypertension | 26.2 |
| Patient 8 | 48 | M | Incidental | 4.0 | Left | chRCC | Nephrectomy | Type 2 diabetes mellitus | 27.8 |
| Patient 9 | 59 | F | Incidental | 4.6 | Left | ccRCC | Nephrectomy | MLH1-related Lynch syndrome (HNPCC2), history of breast cancer, hypertension, parathyroid adenoma, prophylactic hysterectomy | 28.0 |
| Patient 10 | 56 | F | Incidental | 7.5 | Left | pRCC | Nephrectomy | Gallstones | 31.0 |
| Patient 11 | 73 | F | Incidental | 3.0 | Right | ccRCC | Nephrectomy | Asthma, depression, diverticulosis, gastroesophageal reflux disease | 28.6 |
| Patient 12  (tumour right) | 62 | M | Incidental | 2.9 | Bilateral | RO | Active surveillance | Asperger syndrome, hypertension | 30.4 |
| Patient 12  (tumour left) | - | - | Incidental | 5.1 | - | RO | Active surveillance | - | - |
| Group mean  (S.D.) | 64 (8) | - | - | 4.4 (1.6) |  | - |  |  |  |

Supplementary Table 1: Extended patient characteristics. M = male, F = female, RO = renal oncocytoma, ccRCC = clear cell renal cell carcinoma, chRCC = chromophobe renal cell carcinoma, pRCC = papillary renal cell carcinoma, BMI = body mass index.

|  | | | | Phantom-normalised TSC | | | Liver-normalised TSC | | | |
| --- | --- | --- | --- | --- | --- | --- | --- | --- | --- | --- |
| Patient ID | **Histology** | **Tumour diameter** [cm] | **Tumour side** | **Tumour** | **Normal**  **right**  **kidney** | **Normal**  **left kidney** | **Liver** | **Tumour** | **Normal**  **right**  **kidney** | **Normal**  **left**  **kidney** |
| Patient 1 | RO | 3.0 | Left | 102 | 130 | 104 | 40 | 102 | 130 | 104 |
| Patient 2 | RO | 2.5 | Right | 108 | 171 | 168 | 59 | 74 | 117 | 114 |
| Patient 3 | RO | 7.1 | Right | 153 | 102 | 90 | 45 | 137 | 92 | 81 |
| Patient 5  (tumour right) | ccRCC | 5.0 | Right | 125 | 105 | 122 | 53 | 94 | 78 | 91 |
| Patient 5  (tumour left) | chRCC | 3.2 | Left | 72 | - | - | - | 54 | - | - |
| Patient 6 | RO | 5.2 | Left | 185 | 114 | 122 | 74 | 100 | 62 | 66 |
| Patient 8 | chRCC | 4.0 | Left | 69 | 82 | 109 | 38 | 73 | 86 | 115 |
| Patient 9 | ccRCC | 4.6 | Left | 199 | 129 | 140 | 65 | 122 | 79 | 86 |
| Patient 10 | pRCC | 7.5 | Left | 81 | 98 | 86 | 41 | 79 | 95 | 83 |
| Patient 11 | ccRCC | 3.0 | Right | 82 | 71 | 80 | 38 | 88 | 76 | 85 |
| Patient 12  (tumour right) | RO | 2.9 | Right | 163 | 150 | 151 | 71 | 91 | 84 | 84 |
| Patient 12  (tumour left) | RO | 5.1 | Left | 262 | - | - | - | 146 | - | - |
| Group mean (S.D.) | - | 4.4 (1.6) | - | 133 (60) | 115 (31) | 117 (29) | 52 (14) | 92 (27) | 90 (20) | 91 (16) |

Supplementary Table 2: TSC quantification results of kidney tumours and bilateral normal kidney parenchyma. RCC = renal cell carcinoma, TSC = total sodium concentration. Units of TSC are in mM.


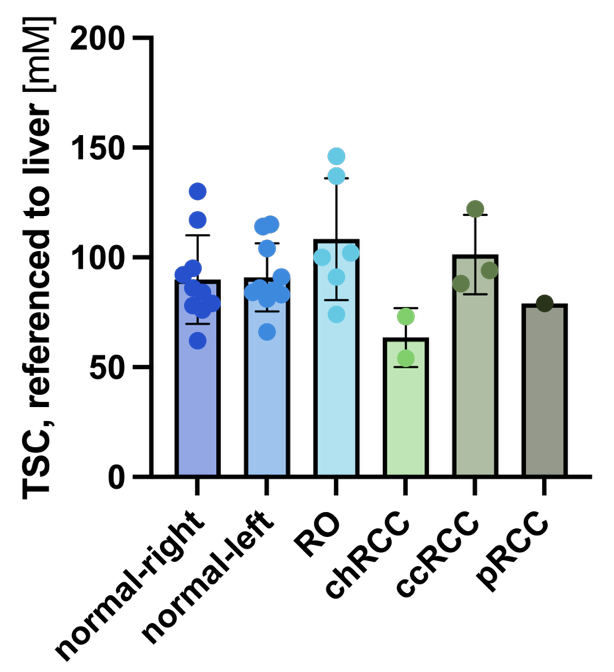


Supplementary Figure 2: Plot representing the B_1_-corrected mean TSC from normal kidney parenchyma and across the kidney tumour subtypes, referenced to liver TSC signal.

|  | | ^23^Na-MRI derived *R*_2_* | | | ^1^H-MRI derived *R*_2_* | | |
| --- | --- | --- | --- | --- | --- | --- | --- |
| Patient ID | **Tumour side** | **Tumour** | **Normal right kidney** | **Normal left kidney** | **Tumour** | **Normal right kidney** | **Normal left kidney** |
| Patient 3 | Right | - | - | - | 16 | 20 | 20 |
| Patient 5  (tumour right) | Right | - | - | - | 28 | 22 | 32 |
| Patient 5  (tumour left) | Left | - | - | - | 30 | - | - |
| Patient 6 | Left | 76 | 49 | 49 | 16 | 22 | 21 |
| Patient 8 | Left | 64 | 41 | 48 | 14 | 21 | 17 |
| Patient 9 | Left | 46 | 57 | 62 | 13 | 20 | 23 |
| Patient 10 | Left | 79 | 40 | 46 | 48 | 18 | 19 |
| Patient 11 | Right | 50 | 60 | 57 | 13 | 22 | 20 |
| Patient 12  (tumour right) | Right | 54 | 48 | 48 | 14 | 17 | 17 |
| Patient 12  (tumour left) | Left | 54 | - | - | 12 | - | - |
| Group mean  (S.D.) | - | 60 (13) | 49 (8) | 51 (6) | 19 (13) | 20 (2) | 19 (2) |

Supplementary Table 3: Quantification of *R*_2_* derived from ^23^Na-MRI and ^1^H-MRI in tumours and normal kidney parenchyma. Units: s^-1^.

|  | | *f*_p_ | | | *D*_t_ | | |
| --- | --- | --- | --- | --- | --- | --- | --- |
| Patient ID | **Tumour side** | **Tumour** | **Normal right kidney** | **Normal left kidney** | **Tumour** | **Normal right kidney** | **Normal left kidney** |
| Patient 6 | Left | 0.034 | 0.075 | 0.051 | 2.264 | 2.106 | 2.144 |
| Patient 8 | Left | 0.085 | 0.057 | 0.044 | 1.832 | 2.542 | 2.606 |
| Patient 9 | Left | 0.097 | 0.006 | 0.019 | 2.071 | 1.826 | 1.933 |
| Patient 10 | Left | 0.045 | 0.007 | 0.022 | 0.970 | 2.061 | 2.066 |
| Patient 11 | Right | 0.154 | 0.066 | 0.059 | 2.279 | 2.066 | 2.028 |
| Patient 12  (tumour right) | Right | 0.028 | 0.060 | 0.091 | 2.082 | 2.115 | 2.047 |
| Patient 12  (tumour left) | Left | 0.028 | - | - | 2.348 | - | - |
| Group mean  (S.D.) | - | 0.067  (0.047) | 0.045  (0.031) | 0.048  (0.027) | 1.978  (0.477) | 2.120  (0.233) | 2.138  (0.239) |

Supplementary Table 4: Quantification of IVIM DWI derived parameters *f*_p_ (in arbitrary units) and *D*_t_ (in × 10^-3^ mm^2^ s^-1^) in tumours and normal kidney parenchyma.
